# Downward migrating microplastics in lake sediments are a tricky indicator for the onset of the Anthropocene

**DOI:** 10.1126/sciadv.adi8136

**Published:** 2024-02-21

**Authors:** Inta Dimante-Deimantovica, Saija Saarni, Marta Barone, Natalja Buhhalko, Normunds Stivrins, Natalija Suhareva, Wojciech Tylmann, Alvise Vianello, Jes Vollertsen

**Affiliations:** ^1^ Latvian Institute of Aquatic Ecology, Riga LV-1007, Latvia.; ^2^ University of Turku, Department of Geography and Geology, Turku 20014, Finland.; ^3^ Daugavpils University, The Faculty of Natural Sciences and Mathematics, Daugavpils LV-5401, Latvia.; ^4^ Tallinn University of Technology, Department of Marine Systems, Tallinn 12618, Estonia.; ^5^ University of Latvia, Department of Geography, Riga LV-1004, Latvia.; ^6^ Tallinn University of Technology, Department of Geology, Tallinn 19086, Estonia.; ^7^ University of Gdańsk, Faculty of Oceanography and Geography, Gdańsk PL-80309, Poland.; ^8^ Aalborg University, Department of the Built Environment, Aalborg 9220, Denmark.

## Abstract

Plastics are a recent particulate material in Earth’s history. Because of plastics persistence and wide-range presence, it has a great potential of being a global age marker and correlation tool between sedimentary profiles. In this research, we query whether microplastics can be considered among the array of proxies to delimit the Anthropocene Epoch (starting from the year 1950 and above). We present a study of microplastics deposition history inferred from sediment profiles of lakes in northeastern Europe. The sediments were dated with independent proxies from the present back to the first half of the 18th century. Regardless of the sediment layer age, microplastic particles were found throughout the cores in all sites. Depending on particles’ aspect ratio, less elongated particles were found deeper, while more elongated particles and fibers have reduced mobility. We conclude that interpretation of microplastics distribution in the studied sediment profiles is ambiguous and does not strictly indicate the beginning of the Anthropocene Epoch.

## Teaser

The epoch of humans—Anthropocene—cannot be precisely defined with microplastic pollution records.

## INTRODUCTION

Microplastics (MPs) are modern pervasive pollutants of anthropogenic origin that pose an environmental and health risk (*1*, *2*). Since the beginning of the production of synthetic polymers in the 20th century, more and more plastic materials with versatile features have been invented and adopted into use (*3*). Cumulative global plastic production reached 8300 million metric tons (Mt) during 2017, and since then, more than 360 Mt of plastics have been produced annually (*3*, *4*). It is estimated that only about 9% of all plastic ever produced is recycled and 12% is incinerated (*3*), leading to the conclusion that over 6000 Mt of waste plastic has the potential to leak into the environment and become incorporated in natural cycles and food chains. Considerable amounts of MPs are found even in arctic deep-sea and rural lake sediments (*5*, *6*), in glaciers (*7*), and in air (*8*) of remote sites. Because of the prominent growth of plastic accumulation across natural environments (*9*–*12*), as well as their potential to persist in geological formations records, MPs are widely suggested as markers of the Anthropocene (*10*–*14*).

In the geological timescale, the Anthropocene (*15*) is currently an unofficial unit proposed to describe the new geological ongoing event/epoch (*16*, *17*), characterized by human altered natural processes. Human activities have left traces in the stratigraphic record during the past few millennia (*18*, *19*). However, qualitatively substantial changes occurred with the “great acceleration” of population growth and industrialization (*20*, *21*). Hence, the Anthropocene Working Group of the International Union of Geological Sciences has suggested the mid-20th century to be the starting point of the Anthropocene Epoch (*22*). This time coincides with the beginning of industrial mass production of plastic; consequently, potential of plastic deposition has been explored among the array of proxies related to the selection of an appropriate Global Boundary Stratotype Section and Point (GSSP). While the Anthropocene Working Group’s suggestion is debated (*23*–*25*), the potential of MPs in terms of time marker for the great acceleration is widely discussed (*9*–*13*, *26*, *27*). Only two suggested GSSP sites included temporal concentrations of MPs (*28*, *29*); however, a broader understanding of the possible coincidence of the earliest temporal occurrence of MPs at the beginning of the base of Anthropocene would be advantageous. Proxies chosen to support GSSP must have global correlation potential in various sedimentary matrices (*13*, *22*, *30*). Precise dating of sediments as young as 1950 presents crucial challenges. Methods that use short-lived natural and artificial atmospheric fall-out radionuclides (^241^Am, ^137^Cs, and ^210^Pb) are most widely used to obtain reliable chronologies for these short timescales and therefore suggested as primary proxies due to their sharp and globally widespread signal. Among these methods, ^210^Pb dating is commonly considered a powerful technique for estimating the age of recently deposited sediments (*31*). However, there are also caveats related to some factors (i.e., water depth, sediment composition, and bioturbation) that influence the vertical activity profiles of ^210^Pb in sediment cores (*32*, *33*). Thus, the dating results should only be accepted if they are consistent with other stratigraphic marker horizons, e.g., ^137^Cs activity peaks, spheroidal carbonaceous particles (SCPs), tephra layers, and well-dated event layers. Potentially, a notable increase in MP concentrations after 1950 would also serve as a marker horizon and ideal addition to the chronological tool box.

However, despite exponential growth in the number of studies on MPs in sediments in general, there are very few studies reporting MP pollution findings from well-dated sediment profiles (*9*, *10*, *29*, *34*–*37*). Hence, the usability of MP profiles as supplementary marker for the Anthropocene remains speculative.

Here, we provide evidence of MP deposition from three lakes (Seksu, Pinku, and Usmas) in Latvia, i.e., the northeastern part of Europe (fig. S1). The lakes represent different degrees of access restrictions due to the status of a specially protected natural area or being a part of the drinking water supply system. There was also a considerable difference among lakes regarding the distance to urbanized areas. The use of well-established dating methods with various proxies (i.e., ^210^Pb and SCP) enabled the evaluation of MP concentration change rates through the 20th century and earlier with precise time control, which is critical in terms of stratigraphy. The presented research measures MP concentration throughout dated lacustrine sediment archives at and below year 1950, demonstrating an exponential growth of MP concentrations introduced into sedimentary records and related to human-induced activities, but not necessarily being time-synchronous. We further discuss variables that might affect MP accumulation in the sediments, and, lastly, we set MPs in a sedimentary context and evaluate MP as a chronostratigraphic marker for the Anthropocene.

## RESULTS

### MP throughout sediments in time and space

**Fig. 1.**
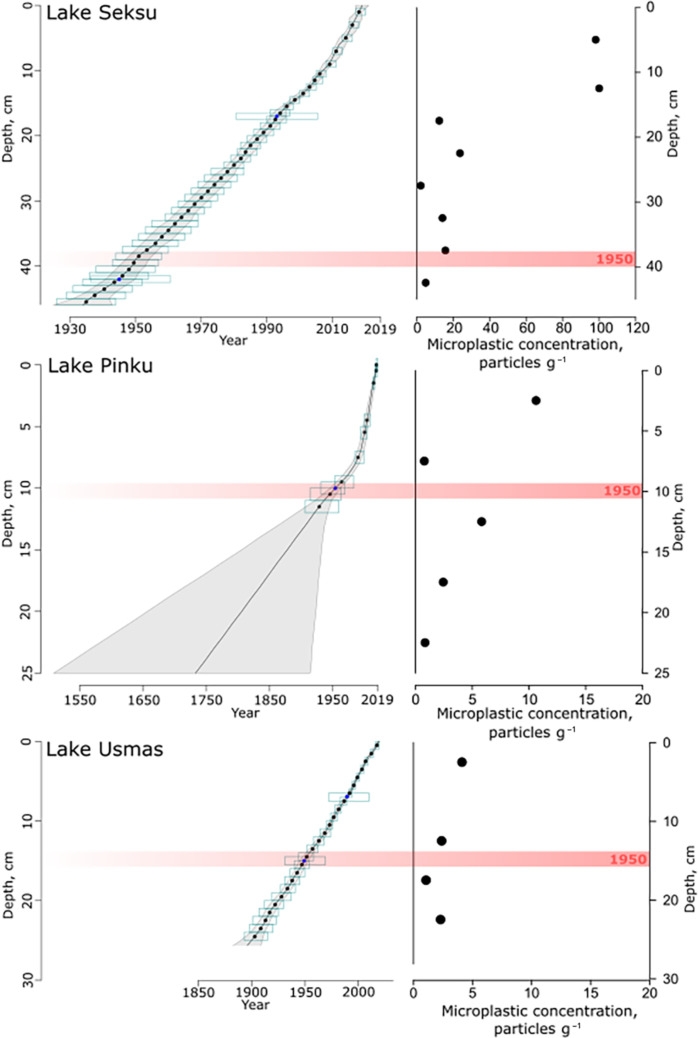
Chronology and associated MP mean concentration for Lake Seksu, Lake Pinku, and Lake Usmas. The black solid line shows the weighted mean ages of all depths, whereas the light gray area indicates the reconstructed 95% chronological uncertainty band. Dates of lead isotope ^210^Pb and SCPs with their error margin age-depth model uncertainties are displayed as light blue boxes. Horizontal red line indicates year 1950.

The combination of ^210^Pb, SCP, and age-depth modeling techniques analyzed with 1-cm resolution provided reliable chronologies for the investigated sediment cores and allowed testing and verifying the potential of MP as a marker horizon. ^210^Pb displayed a regular decrease in activity concentrations with sediment depth and only minor irregularities (Fig. 1 and fig. S2), while SCP indicated concentration pattern, which proves the absence of noteworthy disturbance of the sediment column and allows reliable sediment dating (*38*). The age-depth model for Lake Seksu was almost linear with limited uncertainty indicating a relatively young age of sediments at the basal part (46 cm; age range, 1925–1943; mean age, 1934). Age-depth models for lakes Pinku and Usmas showed reasonable uncertainty until 11 cm (age range, 1921–1949; mean age, 1936) and 25 cm (age range, 1890–1910; mean age, 1900), respectively. Beyond these depths, age was extrapolated because lower parts of the cores were below the dating horizon of ^210^Pb and SCP methods. However, in all three lakes, the age-depth models can be used to reliably establish the boundary between sediment layers older and younger than 1950, which is 39 cm in Lake Seksu, 10 cm in Lake Pinku, and 15 cm in Lake Usmas. Sections of the sediment cores below these depths represent the so-called pre-Anthropocene period, i.e., dating before year 1950 (*13*, *21*, *22*).

**Fig. 2.**
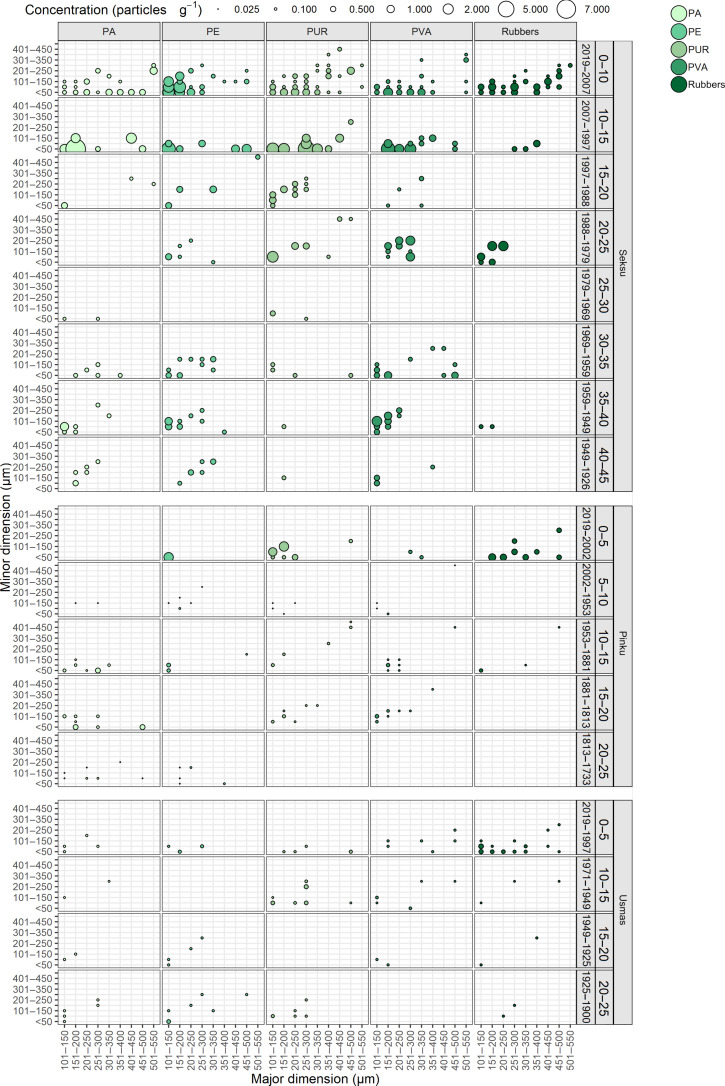
Concentrations and size (the minimum to maximum Feret diameter expressed as minor and major dimension) distribution of dominant MP polymers. PA, PE, PUR, PVA, and rubbers, combined group of BIIR, BR, and SR in sediment core layers and corresponding time period within 5- or 10-cm-depth intervals (identified at right margin) of Lake Seksu, Lake Pinku, and Lake Usmas.

In total, 14 types of polymers and polymer groups were detected in the three lakes—10 types of polymers found in Lake Pinku, 13 found in Lake Usmas, and 14 found in Lake Seksu. The most abundant polymers found at all depths were PA (polyamide), PE (polyethylene), PUR (polyurethane), and PVA (polyvinyl acetate). Group of rubbers [i.e., BIIR (butyl rubber), BR (butadiene rubber), and SR (synthetic rubber)], same as polyester, polypropylene, and PS (polystyrene), were found both in deep and upper layers but not in every layer of the cores. Biodegradable plastics—polylactic acid (PLA) and polyhydroxybutyrate (PHB)—were found occasionally. In all lakes, the highest total concentrations of MPs were detected in upper sediment layers decreasing toward deeper parts of the core. The most common polymers in the topmost 15 cm (representing years 2019–1997) of Lake Seksu were PE and PUR constituting in total 26.6% of all polymers, while in Lake Pinku, 78.9% of all polymers in the upper layer (topmost 5 cm representing time period 2019–2002) were PUR and rubber polymers. Rubbers were the most frequent polymers in the topmost 5 cm (years 2019–1997) in Lake Usmas, using 46.2% of all identified MPs (Figs. 1 and 2 and table S1). The sediment layer 5 to 10 cm (1997–1971) of Lake Usmas was damaged during sample processing and therefore excluded from the further analysis.

There were considerable differences in average concentration of MP throughout the core among lakes, i.e., 2.4, 4.1, and 33.9 particles g^−1^ of dry sediments in lakes Usmas, Pinku, and Seksu accordingly (tables S1 and S2). Lake Seksu is a part of the capital city drinking water system, located in the urban area, protected by access restrictions and fence around, yet showing the highest MP concentrations compared to other two lakes (also protected but located in rural region). Restrictions, however, do not address MP pollution directly; hence, conservation status does not prevent MP pollution. In Lake Seksu, case proximity to the urban environment, i.e., possible MP transport by wind (*39*, *40*) and runoff (*41*), has a more important impact on the MP concentration and composition in the sediments than protection regulations in the lake’s immediate surroundings (*42*).

### Drivers of MP downward movement

According to dry bulk density (BD) data, sediments of Lake Seksu were substantially more loose (0.12 g cm^−3^) than the sediments of lakes Pinku and Usmas (0.68 and 0.44 g cm^−3^, respectively; table S3). The low density of Lake Seksu sediment is related to exceptionally high organic content (on average, 55%) compared to lakes Pinku and Usmas (for both, the average organic matter was 21%) (fig. S4). The linear sedimentation rate was highest in Lake Seksu (5.3 mm year^−1^) and lowest in Lake Pinku (1.3 mm year^−1^).

**Fig. 3.**
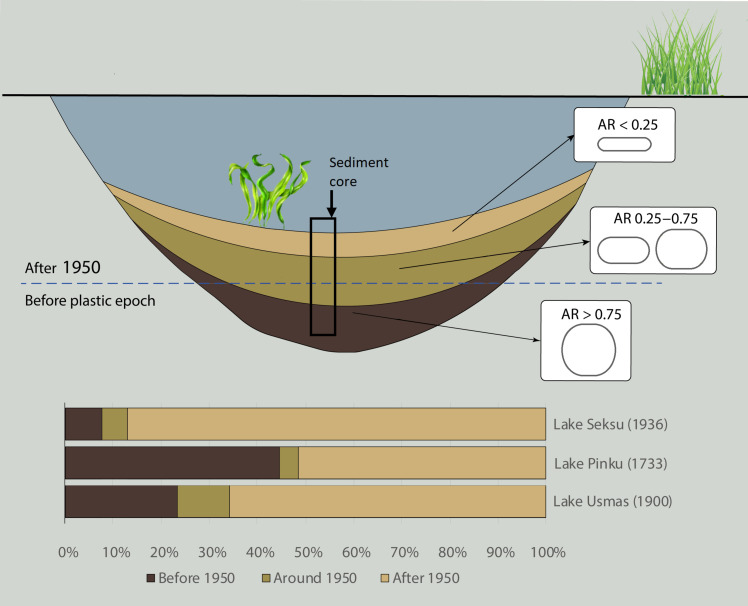
Conceptual representation of MP particles distribution and concentration within lake sediments. General trend of MP particles distribution according to shape (AR, relation of minor dimension to major dimension) and total MP abundance distribution throughout the sediment core before, around and after 1950 in Lake Seksu, Lake Pinku, and Lake Usmas. Year in parentheses represents the age of the sediment core oldest possible layers studied. Note that the basal age of Lake Pinku is extrapolated from the ^210^Pb dating providing a maximum age of 1932 in the depth of 14 cm.

Principal components analysis (PCA) coupled with factor analysis was performed to understand which variables and factors affect the transportation of MP particles into deeper layers. We acknowledged the following variables: sediment depth and age, sediment organic content, BD, relative BD (RBD), MP material density, MP particle size (including major and minor dimension), and shape, i.e., aspect ratio (AR). RBD is equal to the ratio between the BD of each sediment layer and the densest layer of the lake. AR is the relation of minor dimension to major dimension. Hence, we further conventionally categorized particles according to the AR into four classes: <0.25, 0.25 to 0.50, 0.50 to 0.75, and 0.75 to 1. All polymers showed a wide variation of AR at every depth, although the general trend was the greater the AR (less elongated particles), the deeper particles tend to penetrate within a core (Fig. 3 and fig. S5).

**Fig. 4.**
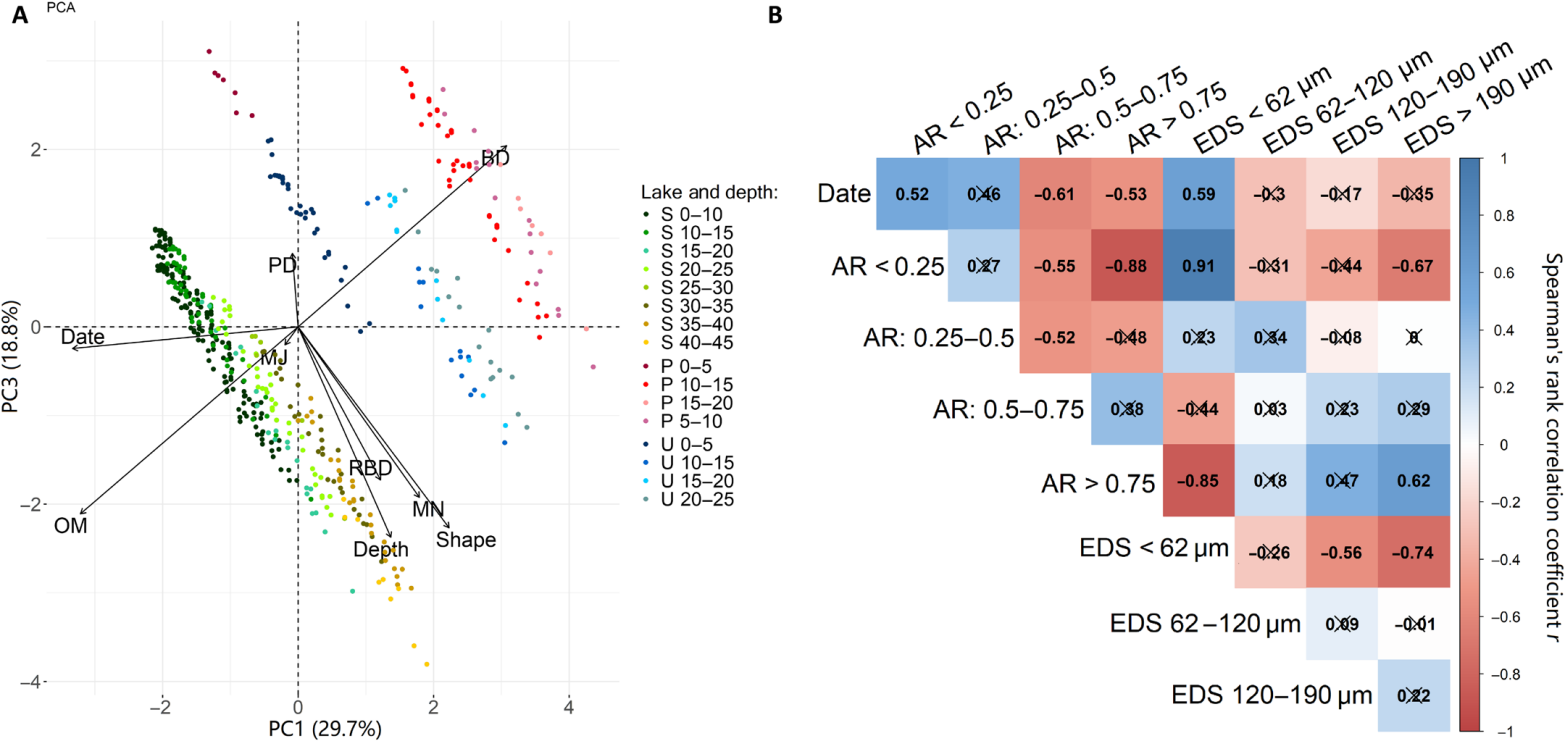
PCA and Spearman’s rank correlation matrix. (**A**) The selected (see Materials and Methods) principal component 1 (PC1) and PC3 together explain 48.5% of variations. Variables: date, dating of sediment layer; OM, organic matter content; depth, depth of sediment layer (in centimeters); shape, AR of particle minor dimension versus major dimension; MN, particle minor dimension; MJ, particle major dimension; PD, density of polymer; BD, bulk density of sediments; RBD, relative bulk density of sediments (average BD of a core layer to the densest layer of the lake). P, Lake Pinku; S, Lake Seksu; U, Lake Usmas. (**B**) Spearman’s rank correlation matrix. Correlation for the composition of four particle groups with different values of AR: Group 1 is AR < 0.25; group 2 is 0.25 ≤ AR < 0.5; group 3 is 0.5 ≤ AR < 0.75; and group 4 is AR ≥ 0.75 and relative dating of the sediment core layer in pulled data of lakes Seksu, Pinku, and Usmas. The coefficients (Spearman’s rho) are colored on the basis of their sign and strength (the legend of continuous color scale positioned on the right side), and insignificant correlations (*P* > 0.05) are crossed out.

The individual AR of the MP particles was the only variable correlating with the depth (Fig. 4A). The PCA analysis showed that transportation of MP into the deeper layers was neither affected by these geological variables as BD of sediments and organic matter content nor MP density and particle size.

Spearman’s rank correlation analysis of the pooled data from the three lakes showed a strong positive correlation (Fig. 4B) between the age of the sediment layer (date, as a proxy of depth for pooled data of three different lakes) and the proportional abundance of the most elongated particles (AR < 0.25) found in the layer, as well as a strong negative correlation with the proportional abundance of the least elongated particles (0.5 to 0.75 and 0.75 to 1). Equivalent spherical diameter (ESD) is a particle size descriptor that allows the comparison of irregularly shaped objects via the calculated diameter of a sphere of equivalent volume. Only the proportional abundance of the smallest fraction with ESD below the first distribution quartile (ESD < 62 μm) showed a strong positive correlation with the age of the sediment layer, but the result should be interpreted with caution because, according to the ESD equation, a smaller minor dimension for the same major dimension results in a lower ESD, which may mean that the particle has a lower AR but not the size itself. The results indicate that particle shape plays an important role in the ability of a particle to be mobile within the sediment column.

## DISCUSSION

Our results are comparable with other findings summarizing data on polymers occurring in fresh waters and estuaries (*43*, *44*). Nevertheless, we did not compare exact MP concentrations per sediment unit with previous studies since, in our results, the actual MP concentration in the lake sediments might be at least twice or even three times higher. This is due to the fact that our study recovery tests revealed, on average, 32.6% recovery reflecting the heavy purification protocol involved with many procedural steps, i.e., the number of sample transfers during sample purification affects the recovery rate (*45*).

Nevertheless, there is no doubt that sediments serve as an important sink for MP in fresh waters. The increasing trend of MP concentration toward younger (surface) sediments is a well-established and understood finding related to accelerating mass production of plastic materials (*10*, *11*, *28*, *46*). The Lake Pinku concentration shows similar trend with increase from 0.8 to 10.6 particles g^−1^ between the two topmost samples (layers dated 2002–1953 and 2019–2022, respectively). In Lake Seksu, MP concentration peaked above the sediment layer 15 to 20 cm dating back to 1997–1988, when the concentration increased from 11.9 to 100.1 particles g^−1^ (fig. S3). This indicates that there is no elbow point reflecting MP pollution rise after 1950s. Instead, a clear elbow in Lake Seksu core reflects the beginning of the past two decades coinciding with doubling of annual production rate of plastics in 2002 compared to 1989 (from 100 to 200 Mt) (*47*). This is consistent with the results by Turner *et al.* (*9*) and Brandon *et al.* (*10*) reporting notable increase in MP concentrations around the time. Local evolvement can also be linked to MP concentration changes, i.e., Long *et al.* (*48*) showed MP concentration changes to be reasonable because of changing national policies and strengthened environmental legislation same as Waste Water Treatment Plants introduction. Lake Seksu MP concentration rapid increase coincides with time when country and society transformed from Soviet planned economy into free market and economic society that, in turn, could create more potential locally based sources of MP.

Still, a considerable part of the total MP pollution is accumulated in the sediments deposited before 1950 according both to our and other studies with well-dated sediment records (*9*, *10*, *29*, *34*–*37*). These findings are often treated as artifacts of sample processing. For example, the Santa Barbara basin record, where a varved sediment structure denotes the absence of physical mixing, shows a clearly increasing trend in fragments and films starting from sediments older than 1950 [see supplementary data from the study of Brandon *et al.* (*10*)]. On the contrary to Brandon *et al.* (*10*), in our study, positive and negative controls were used; hence, the increasing concentration of MPs starting from pre-1950 level is unlikely caused by artifact (i.e., contamination during samples preparative procedure). Same conclusions can be derived from other studies (*9*, *29*, *34*–*37*).

Among others, biodegradable plastics such as PLA and PHB were also recorded from our samples. The breakthrough of biodegradable plastics took place in the 1990s (*49*). Depending on the environment, temperature, size, and shape of the particles, the degradation in the environment can take several years (*50*, *51*). Hence, particles found in the lake should not be older than the past decade. Still, in our study, PLA and PHB were found in rather old sediment layers, e.g., 1925–1900 (Lake Usmas), 1953–1881, and 1813–1733 (Lake Pinku).

Therefore, we suggest that these findings show a true natural phenomenon, unambiguous downward movement of MPs in sediment profiles. Several mechanisms can transport MP particles deeper—core smearing during sampling, sediment reworking through resuspension, or turbidity flows and bioturbation (*52*). There is no evidence of substantial particle movements resulting from coring in our results nor in the literature (*53*). The volume of material close to the liner walls is comparatively low compared to the rest of the sediment core sample volume. Moreover, large cores tube diameter that we used, with short penetration depth and loose surface sediments, are considered less prone for core smearing (*54*). Because of precise and consistent time control of the investigated sediment cores, sediment reworking including bioturbation can be excluded. In Lake Pinku, more than 40% of MPs occurred below the sediment layer of the 1950s, and noticeable MP concentrations are found in deeper layers of lakes Seksu and Usmas compared to the total MP concentration throughout the core and to our contamination controls. The high number of MP particles below 1950 level supports our argument that occurrence of MP downward movement cannot be explained as an artifact of sample preparation or sediment coring. Hence, we question the use of pre-1950 sediments as core processing contamination controls in MP studies.

Downward migration through low-density organic sediments is described in cryptotephra studies, explained by the higher density of the tephra shards (1 to 2 g cm^−3^) leading to particle sinking through soft, porous sediments until higher density facies (*53*, *55*). MP downward movement is previously related to low sediment density and small particle size of MPs as well as large grain size of the sediment matrix (*56*). Pore water movement within porous sediments is also discussed as a possible mechanism (*57*). The density of most plastic materials is considerably lower than that of tephra shards, and our analyses did not support the density-driven penetration of MP particles as a primary explanation. PE, which has a density of <1 g cm^−3^, was one of the most frequent polymer types found below 1950 level. Leiser *et al.* (*58*) have observed that aggregation to iron-organo flocs causes faster burial of PE particles and downward migration within sediment column up to 11 cm in unconsolidated muddy lake sediments. This mechanism can well have an important role. Cycles of anoxic and oxic conditions at the bottom as a consequence of stratification and mixing in the eutrophic dimictic lakes favor floc formation.

Leiser *et al.* (*58*) also suggested that the consequent gas formation within the anoxic sediment following the breaking of the flocs could cause PE particles to sink further within the sediment column. This mechanism can have importance in the burial of other plastic polymers as well.

Zheng *et al.* (*59*) suggested the deeper the sediments, the smaller ESD of the MP particle. Same was claimed by Waldschläger and Schüttrumpf (*60*), i.e., infiltration depth increases with decreasing ESD of the MP particle; moreover, the infiltration depends on ratio between diameters of the MP particles and the sediment fraction. Our results showed that less elongated particles tend to penetrate deeper into sediments, while elongated particles (AR = 0.5 to 1) are more likely to remain in the upper layers. There is also a trend for larger (according to major dimension) particles to be located in layers more toward the surface (Fig. 2 and fig. S6), suggesting that migration might be size/shape-selective to a certain extent. This is in line with the finding of Leiser *et al.* (*58*), where smaller PE particles incorporated with iron-organo flocs penetrated deeper in the sediment column, and Hinata *et al.* (*28*) who studied rather large particles (355 μm and above excluding fibers) and did not find any MP in samples older than 1958.

Because rounded particles show stronger tendency to penetrate deeper in the sediment profile, our results agree with previous study by Bancone *et al.* (*14*), suggesting that fibers have more potential as dating tool than fragments.

Material characteristics such as hydrophobicity could further influence MP migration. Gao *et al.* (*56*) found the plastic materials with higher contact angle (hydrophobic > 90°) penetrating in smaller depths within soil. Within the aquatic environment, decreased water content occurs deeper in the sediments in contradiction to increasing humidity deeper in the soil formations. Hence, this behavior is expected to oppose findings from soil studies. Water column and wet surface sediments may drive hydrophobic particles downward within the sediment profile during the compaction process. PVA with contact angle around 60° (*61*) is less represented deeper within sediment profiles despite their common occurrence in surface sediments and larger density compared to hydrophobic PE (96° or more) (*62*) with density lower than that of water yet being most frequent plastic material observed in the layers older than 1950s. Being one of the first plastic materials to generalize, PE particles have likely prevailed in nature for a longer time that can be reflected in the deeper occurrence of PE materials.

To give a synopsis, we raise an important concern related to the use of MPs as an additional stratigraphic marker for the post-1950 great acceleration. Although all these records show great acceleration regarding MP accumulation rates and concentrations equivalent to growing global net plastic production mass, the first appearance of MPs in the sedimentary archives does not reflect the onset of plastic production and hence fail to reflect the onset of Anthropocene.

Because of the MPs downward movement to varying depths through unconsolidated sediment profiles, the occurrence of MPs cannot directly be used as a time-synchronous marker horizon for the Anthropocene. The migration depth likely depends not only on particle shape but also on sediment porosity and sedimentation rate. Hence, especially organic-rich low-density sediments and locations with low sedimentation rates should be investigated with a severe caution. In addition, careful dating with ideally several independent and robust dating methods should be widely introduced. Consequently, MP becomes not rational as a chronostratigraphic marker due to low confidence and high costs. Further experimental laboratory and field studies are required to fully understand the drivers of MP in the sediments vertical distribution, same as observed phenomena that need to be studied and confirmed in other environmental matrices apart from marine and lacustrine sediments, i.e., in soil and fluviatile systems.

## MATERIALS AND METHODS

### Study site description and sampling

Sediment cores (one core per each lake from the deepest part of the lake) were taken from three lakes in Latvia (fig. S1). Lake Pinku is an oligotrophic/mesotrophic lake located in a glacier depression. It is of high water quality, and since 2004, the lake and its surrounding area has been a part of a protected nature park. The nearest regional motor road is approximately 600 m away. The lake has a surface area of 29 ha, a length of 1.3 km, a greatest width of 0.3 km, an average depth of 4.3 m, and a greatest depth of 20 m (*63*). There are one inflow ditch and another outflow ditch. The core (25 cm in length) was taken in August 2019 (latitude: 56°59′58.20″, longitude: 21°41′14.72″) by a 5.2-cm–inner diameter Kajak corer.

Lake Usmas is among Latvia’s largest lakes, with a surface area of 3469.2 ha and a water volume of 0.19 km^3^. It is mesotrophic/eutrophic lake, located in a glacier depression, and has several islands. Part of the lake (outside sampling area) belongs to a nature reserve. Apart from this area, the lake is a famous destination for recreational activities. The major motor road is approximately in 680 m in distance. The lake is also surrounded by several local roads. The lake has a length of 13.5 km, a greatest width of 6.2 km, an average depth of 5.4 m, and a greatest depth of 27 m. There are more than 10 inflow rivers and ditches and one outflow river (*64*). The core (25 cm in length) was taken in August 2019 (latitude: 57°13′45.32″, longitude: 22°10′26.97″) by a 5.2-cm–inner diameter Kajak corer.

Lake Seksu is a eutrophic, small (surface area of 7.9 ha, length of 550 m, and greatest width of 350 m) and shallow (average depth of 2.5 m and greatest depth of 6 m) lake located in the vicinity of Latvia’s capital city, Riga. Major motor roads are located on both sides of the lake (the nearest—one of the most extensively used major motor roads in the country is in approximately 850 m in distance). Inland dune forests mainly surround the adjacent catchment. The lake is part of the drinking water supply system enriching the groundwater level near the drinking water pump station. Because of that, access to the lake is limited, and a fence surrounds the lake. Water in the lake was artificially replenished between 1953 and 1965 from close to the eutrophic lake to increase the water level. The lake has no outflow, and there is an inflowing ditch (*65*). The core (45 cm in length) was taken in February 2019 (latitude: 57°2′10.35″, longitude: 24°21′6.73″) by an 8-cm–inner diameter Kayak/HTH gravity corer.

The sediment cores were divided in the field into 1-cm sections, placed in specially prepared (washed and muffled) glass jars, and covered by aluminum foil and metal lids. Samples were stored in a cold room. Part of the material (4 to 5 cm^3^) of each 1-cm sediment section was dried and used for ^210^Pb and SCPs dating as well as for dry weight estimation and chemical and physical analyses.

### MP sample preparation and analysis

MP samples were prepared as a consecutive section with 5-cm intervals of mixed subsamples of the core. However, because of the very high water content of the topmost samples of Lake Seksu, the upper sample of the core consisted of 10 cm. Samples purification was done by applying a multistep treatment method (fig. S7) adapted from (*66*–*71*).

Samples were analyzed using Fourier transform infrared microspectroscopy (μFTIR) (Perkin Elmer Spotlight 400). A subsample of the total 5-ml sample (at least 0.5 ml) was taken using a capillary glass pipette (microclassic, Brand GmbH, Germany) and filtered through 11-mm by 11-mm Si filter (Fraunhofer Institute for Reliability and Microintegration, Germany). In case that sample contained low number of particles and for the blank samples, the total volume of the sample (5 ml) was analyzed. Filters were left to dry for 12 hours at room temperature. The analysis was done applying μFTIR imaging technique in transmission mode in a spectral range of 4000 to 750 cm^−1^ at 8 cm^−1^ resolution. The whole surface of the filter was scanned, and infrared spectra of particles were obtained. The polymer assignments of the analyzed particles were based on comparison with a FTIR spectral library developed at Tallinn University of Technology and in Leibniz Institute for Polymer Research Dresden. Spectral libraries comprise spectra of artificial polymers and natural organic and inorganic materials. The threshold for accepting the match was set to 70%, but all matches were verified by the operator as well. Cross-validation of measured spectra between Tallinn University of Technology and Aalborg University, Department of the Built Environment was made. At the same time, a light microscope image of the inspected particles was produced for visual inspection and size determination (*72*). Plastic particulates were also identified and categorized by size class for greatest length and width dimensions (major dimension is the longest side of the particle, and minor dimension is the shortest side of the particle). Principles of measuring particles were taken from the protocol of Sun and Liu (*73*) for measuring cell bio volume and surface area. We followed only measurement instructions from this work. To present measurements, we used 50-μm intervals from 51 to 550 μm. One size step (50 μm) below and above fractioning sieves size was chosen to recover fibers as much as possible.

### Quality control and blank tests

To avoid airborne and cross-contamination or unintentional loss of MP particles, several precautionary measures were taken. All equipment that was used for samples storage and purification or came in contact with the samples in any other way was made from glass, polytetrafluoroethylene, or metal when possible and was thoroughly rinsed with filtered Milli-Q water before use. The polymer spectrum of all plastic materials, which were in contact with samples and not possible to replace with glass or other alternatives (e.g., sediment corer, gloves, bottle corks, etc.), was recorded and excluded from further polymer analysis. Cotton laboratory coats of a specific color (green) and blue or green nitrile gloves were worn while working with samples. The samples purification was performed in the laminar flow cabinet. The same beaker was used when possible throughout the purification process for each sample, rinsing it with filtered Milli-Q water between each purification step. Samples were covered with aluminum foil when not processed or when placed in the shaking-heating bath located in the fume hood. All reagents were filtered through a glass fiber filter (pore size, 1.2 μm).

Laboratory and field blanks were run parallel to real samples, applying the same processing steps to collect information about sample contamination degree during sample purification. The air background sample showed contamination of 3.4 fibers hour^−1^ (only, no fragments) on average. Procedural and field blanks contained mostly viscose (65.7 to 100% of all polymers found in the blank samples), most likely from the clothing. Viscose was consequently removed from the further data analysis. Other polymer particles were detected only in very low numbers (few particles per blank sample). Recovery tests were performed with triplicate lake sediment samples spiked with standardized 100 red ⌀100-μm PS beads and a density of 1.05 g cm^−3^ (Sigma-Aldrich, product no. 56969-10ML-F). The spiked samples were processed as described in the protocol for sediment samples. Extracted beads were easily identified because of their distinct appearance and were counted under a light microscope Leica DM400 B light-emitting diode.

### Core chronology sample preparation and analysis

The activity of total lead isotope ^210^Pb was determined indirectly by measuring polonium isotope ^210^Po using alpha spectrometry. Freeze-dried sediment samples of 0.2 g were spiked with a ^209^Po yield tracer and digested with concentrated nitric acid HNO_3_, perchloric acid HClO_4_, and hydrofluoric acid HF at a temperature of 100°C (CEM Mars 6 microwave digestion system, USA). Next, the solution was evaporated with 6 M hydrochloric acid HCl to dryness and then dissolved in 0.5 M HCl. Polonium isotopes were spontaneously deposited within 4 hours on silver discs (*74*). After deposition, the discs were washed with methanol and analyzed for ^210^Po and ^209^Po using a 7200-04 APEX Alpha Analyst spectrometer (Canberra, USA) equipped with PIPS A450-18AM detectors. The samples were counted for 24 hours. A certified mixed alpha source (^234^U, ^238^U, ^239^Pu, and ^241^Am; SRS 73833-121, Analytics, Atlanta, GA, USA) was used to check the detector counting efficiencies, which varied from 30.9 to 33.9% for the applied geometry. Two blank samples were analyzed with each sample batch to additionally verify the quality of the chemical procedure. The excess ^210^Pb (i.e., total ^210^Pb activity minus the supported ^210^Pb activity) estimated from the lower sections of the cores (*31*) was used for dating.

According to the black carbon combustion continuum model of Hedges *et al.* (*75*) and Masiello (*76*), SCP only form during industrial fuel combustion at high temperature (>1000°C). A load of SCPs along the sediment sequences were estimated and followed the methodology of Rose (*77*). The sediments were subjected to sequential chemical purification using H_2_O_2_, potassium hydroxide (KOH), and HCl to remove organic material, silicates, and carbonates, respectively. *Lycopodium* tablets (*78*) with a known amount of spores were added as markers allowing estimation of SCP per sample. Slides for the microscope were prepared afterward, and all SCPs within the whole slide were counted under a light microscope at 400 times magnification. Identification criteria for SCP counting followed Rose (*79*). The concentrations of SCP were calculated as a number of particles per 0.2 g of dry mass of sediment. Across all sites, the record of SCP starts at the beginning of the 20th century and rapidly increases since the 1950s. The peak in SCP emissions in Latvia occurred in 1982 ± 10 (*38*). After rapid increase and peak in SCP concentration followed a decline that coincided with the collapse of the Soviet Union when numerous manufactures and air pollutants halted their production in the Baltic region. The SCP occurrence pattern in our study mirrors worldwide SCP pattern change (*80*) following the fuel combustion pattern: 1950, the rise of SCP; specific to Latvia 1982, the peak of SCP; specific to Latvia 1991, the decrease in SCP (fig. S2).

The dating was performed with 1-cm resolution except that for the topmost unconsolidated part, a 2-cm sediment sample was used. Results of the ^210^Pb dating with the CFCS (constant flux constant sedimentation) model and the SCP analyses were used to build an age-depth model using the Clam deposition model (*81*) package with a 95.4% confidence level in the R environment (*82*). The mean weighted value of the modeled age was selected. While ^210^Pb dating provided the basal ages of the cores for Lakes Usmas and Seksu, it provided a maximum age of 1932 CE for Lake Pinku at the depth of 14 cm. Thus, the dates below 14 cm in depth are extrapolated assuming constant sedimentation to reach 1733 CE. While the uncertainty for the ages below 1932 at Lake Pinku is large, our dating confirms the critical age at 1950 with high certainty.

### Loss-on-ignition samples preparation and analysis

The dry weight of 1-cm-thick subsamples with a 1-cm^3^ volume was determined after oven-drying at 105°C until constant weight. The organic matter content of the sediment was determined by loss on ignition (LOI) at 550°C for 4 hours. The carbonate matter was calculated as the difference between the LOI at 950°C and the LOI at 550°C. Because the weight loss after 950°C is the amount of CO_2_ evolved from carbonate minerals to get the actual percent of CO_3_, the weight after 950°C combustion was multiplied by 1.36 (*83*, *84*). Dry BD (in grams per cubic centimeter) was estimated on the basis of the LOI for all samples. Noncarbonate siliciclastic matter, here referred to as minerogenic matter content, was obtained by subtracting organic and carbonate matter from the total sample weight after final combustion. All values for organic, carbonate, and mineral matter are expressed as percentages.

### Data analysis and statistical assessment

Particles were categorized into size ranges (with intervals of 50 μm) up to size class 51 to 550 μm considering two diametrical dimensions (major and minor dimension). Plastic densities data were obtained from existing databases. If density range was rather wide, e.g., for PA and PUR that can be made in a variety of densities and hardnesses, particular polymers were not included in the density analysis (*85*, *86*). PCA was applied to the data to understand which variables and factors are driving the transport of particles into deeper sediment layers. To include information about particle elongation (or shape) on a continuous scale, the AR (*87*) was calculated for each analyzed particle. AR is a shape descriptor defined by the ratio of the minimum to maximum Feret diameter (*87*)

$$\text{AR}=\frac{X_{\text{Feret min}}}{X_{\text{Feret max}}}$$

(for easier interpretation of the results, the AR is expressed in decimal fractions; in the reviewed literature, this form of expression is used less often than the length-to-width ratio). In addition, a variable such as RBD has been added to the dataset to normalize substantial variations in bulk density between sediments of different lakes. The most representative dimensions of PCA were determined according to the commonly used methodology, implementing Kaiser-Guttman criterion (*88*) together with the scree plot of eigenvalues and biplot visualization output; therefore, PC1 (29.7%) and PC3 (18.8%) were selected as the best explanations of variances (PC2 was equal to 19.3%, but visualization of individuals’ groups was weaker). PCA output, variable coordinates, quality of the factor map COS2, and variable contribution can be found in table S3.

Spearman’s rank correlation test between age (date) of sediments, AR, and ESD was performed on the entire dataset of the pooled data of the three lakes to increase data length and include data on Lake Usmas, which was not sufficient (only four data lines) for a separate analysis. Thus, it was not possible to use layer depth as a correlating variable for the pooled data due to the different BD and of the sediments. Instead, the age of the sediments was used as a measure of depth, normalized to BD. The verified relationships were considered statistically significant at *P* < 0.05. For the analysis, the AR was conventionally divided into four groups: Group 1 is AR < 0.25; group 2 is 0.25 ≤ AR < 0.5; group 3 is 0.5 ≤ AR < 0.75; and group 4 is AR ≥ 0.75, to show differences in proportional composition of those groups in each sediment layer. The ESD (*89*), as a standardized particle fraction index, was calculated according to the formula

$$\text{ESD}={\left(AC^{2}\right)}^{\frac{1}{3}}$$

where *A* is a particle’s major dimension (or *X*_Feret max_) and *C* is a minor dimension (or *X*_Feret min_). Similarly to AR, to see how the proportion of particle fractions varies in different sediment layers, ESD was divided into four groups according to the lower quartile, median, and upper quartile of the ESD distribution: Group 1 is ESD < 62 μm; group 2 is 62 μm ≤ ESD < 120 μm; group 3 is 120 μm ≤ ESD < 190 μm; and group 4 is ESD ≥ 190. Spearman’s rank correlation test was chosen because the AR proportional composition variables were not normally distributed; moreover, date is an ordered variable.

Elbow points of MP concentrations in sediment cores were calculated by means of akmedoids package (version 1.3.0) in R environment (*82*). Analysis was done for lakes Pinku and Seksu only. The number of data from Lake Usmas was not sufficient for this analysis. Data exploration, artworks, and statistical analyses were performed using R software for Windows, release 4.0.3 (*82*) and GNU Image Manipulation Program (GIMP), release 21.10.30 (*90*).
