# Supplementary material for: Downward migrating microplastics in lake sediments are a tricky indicator for the onset of the Anthropocene
Source: Sci Adv. 2024 Feb 21;10(8):eadi8136. doi: 10.1126/sciadv.adi8136 (PMC10881056; doi:10.1126/sciadv.adi8136)
Supplement: Supplementary file 1 — Figs. S1 to S7 Tables S1 to S3 [file sciadv.adi8136_sm.pdf]

Science Advances
AAAS

Supplementary Materials for

Downward migrating microplastics in lake sediments are a tricky indicator for the onset of the Anthropocene

Inta Dimante-Deimantovica *et al.*

Corresponding author: Inta Dimante-Deimantovica, inta.dimante-deimantovica@lhei.lv;
Saija Saarni, saija.saarni@utu.fi

Sci. Adv. **10**, eadi8136 (2024)
DOI: 10.1126/sciadv.adi8136

This PDF file includes:

Figs. S1 to S7
Tables S1 to S3

Supplementary Materials

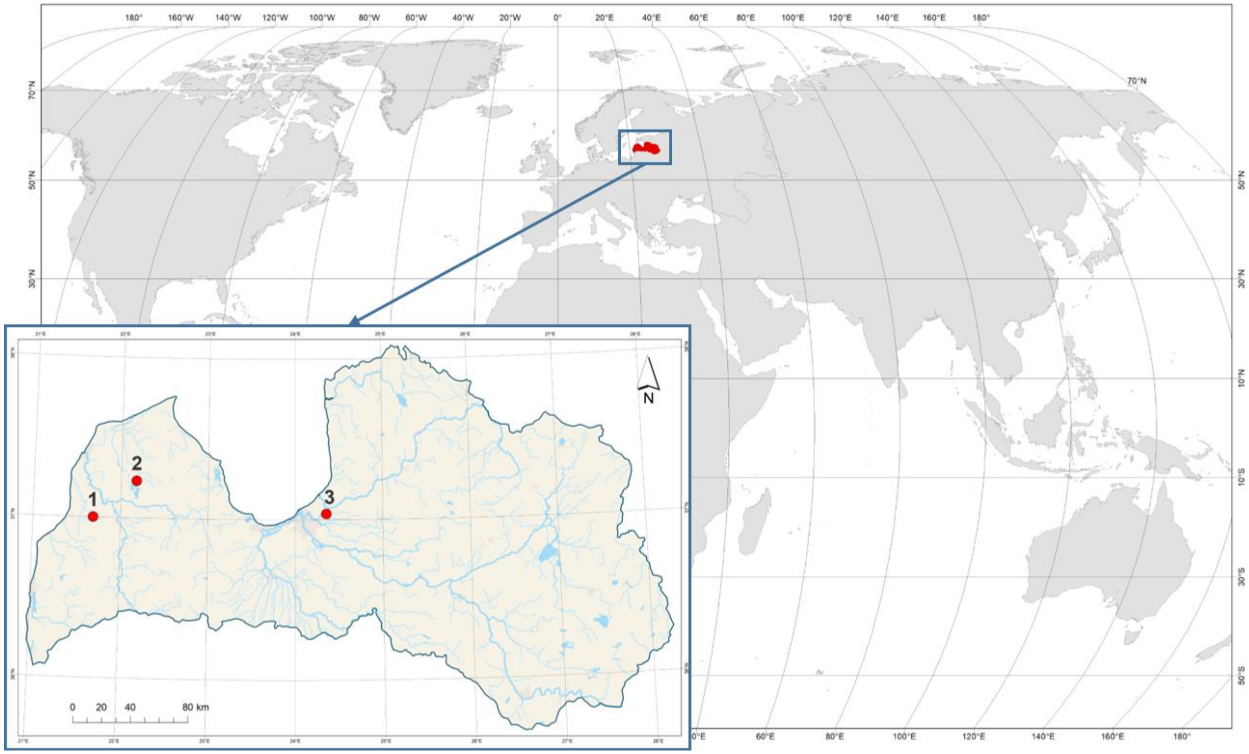

Fig. S1. Location of study sites. Sampling in three Latvian lakes, northeastern Europe: 1 - Lake Pinku, 2 - Lake Usmas, 3 - Lake Seksu. Map is generated using ArcMap 10.6.1.

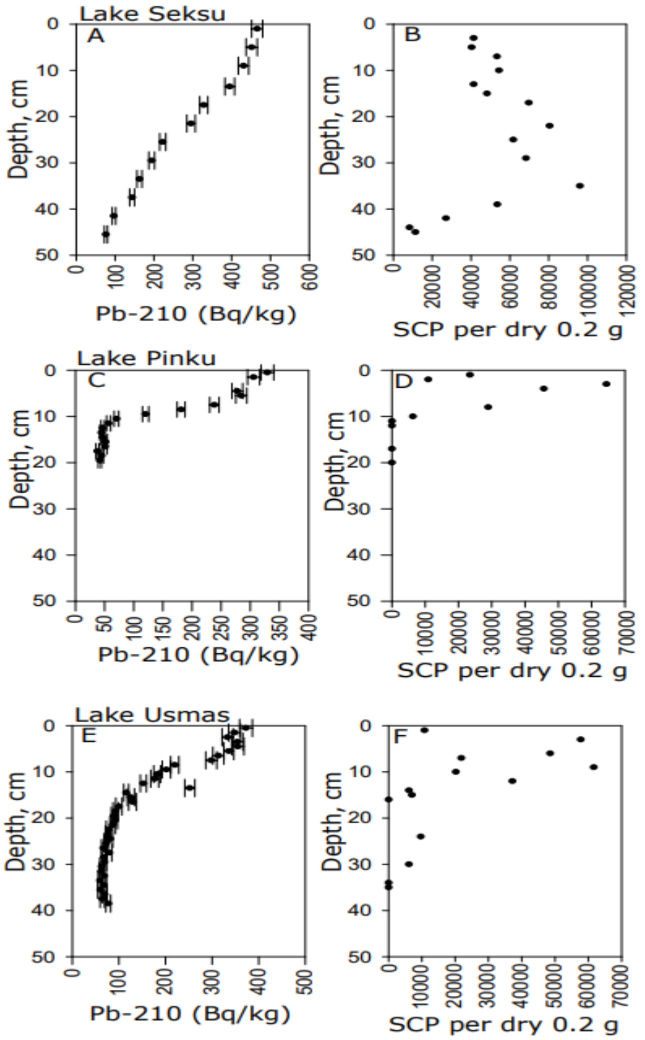

Fig. S2. Downcore distribution of lead isotope 210Pb and spheroidal carbonaceous particles (SCP) in Lake Seksu (A - B), Lake Pinku (C - D) and Lake Usmas (E - F) sediments.

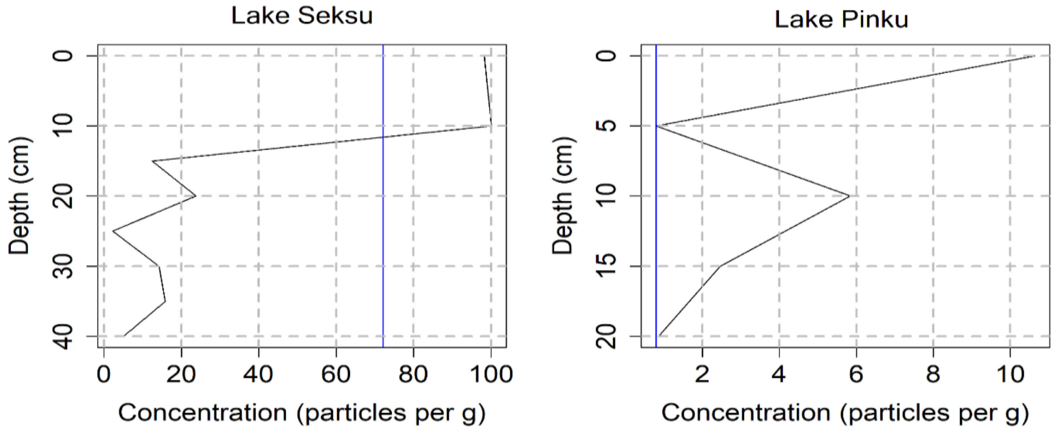

Fig. S3. Meaningful elbow points calculated for Lakes Seksu and Pinku (there was too little data of lake Usmas). The elbow point at Lake Seksu was pointed at a depth of 10-15 cm, where the concentration of microplastics (MP) was 100.1 particles·g^{-1}. The elbow point at Lake Pinku was found at a depth of 0-5 cm referred to the MP concentration of 0.8 particles·g^{-1}.

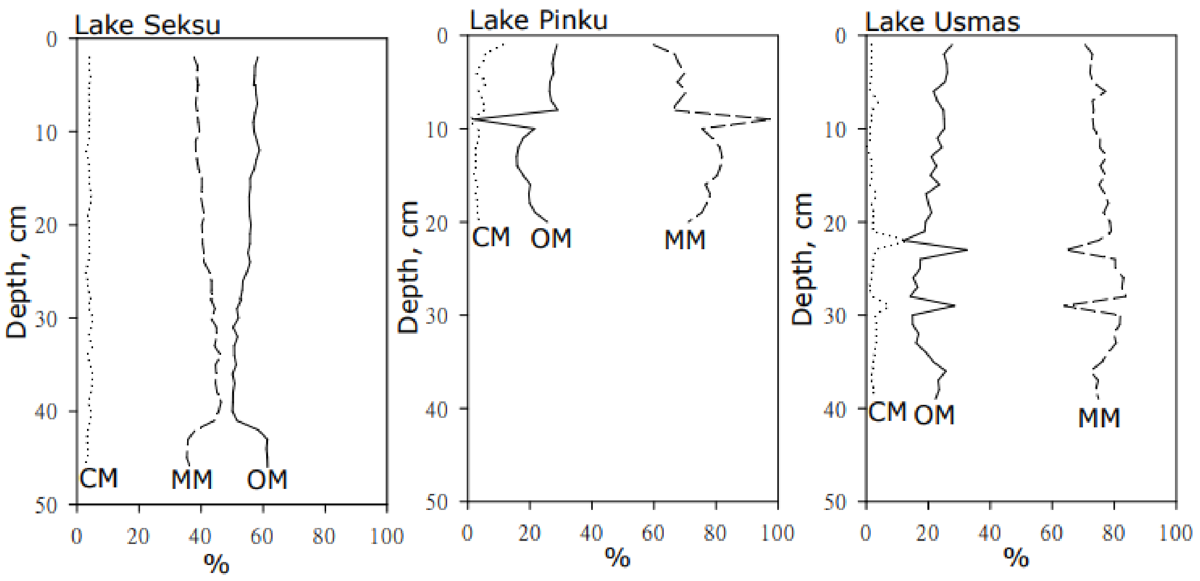

Fig. S4. Organic (OM), mineral (MM) and carbonate matter (CM, %) content in Lake Seksu, Lake Pinku and Lake Usmas sediment.

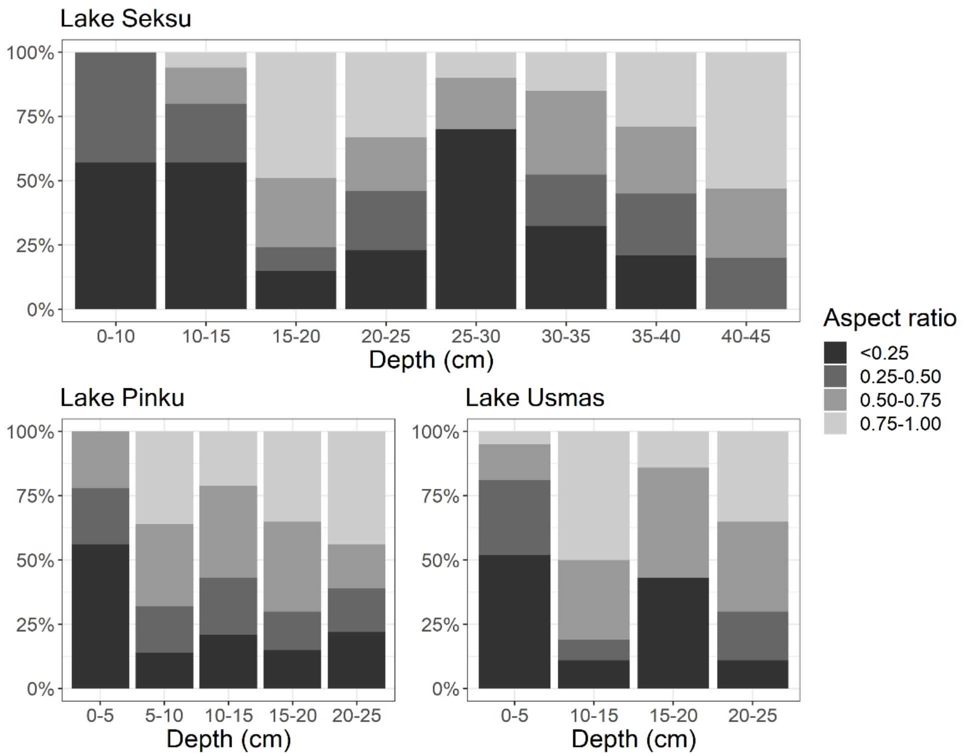

Fig. S5. Particles aspect ratio (minor vs major dimension) throughout the cores in lakes Pinku, Usmas, Seksu.

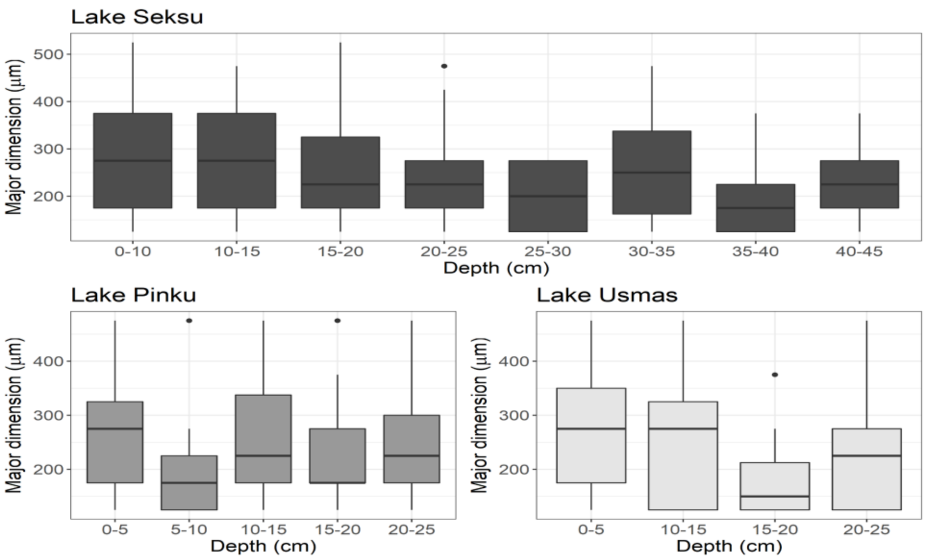

Fig. S6. The boxplot of distribution of particles described by major dimension between different depths of sediments in lakes - Seksu, Pinku and Usmas.

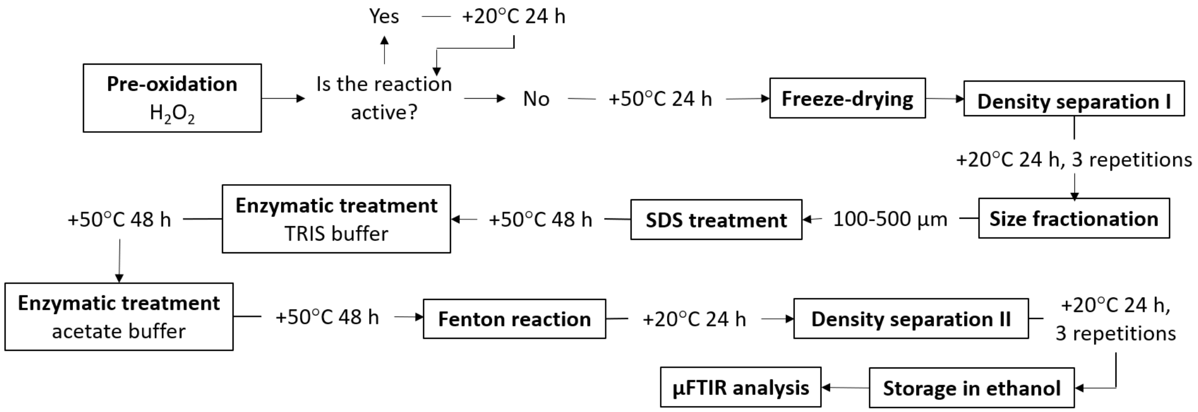

Fig. S7. Microplastic sediment samples preparation/purification scheme. First, 30 % hydrogen peroxide (H_2O_2) was added to the samples in proportion 1:2. Samples were incubated at room temperature (+20 °C) or in a shaking bath (+50°C, 100 rpm) for 24 h - 48 h depending on the treatment step. Density separation was performed in a separatory funnel with heavy-density liquid (sodium polytungstate, density 1.75 kg L^{-1}), followed by size fractionation using 100 µm and 500 µm stainless steel sieves. Only the size fraction 100-500 µm was further used. The relatively high lowest size border (100 µm) was used to remove a considerable amount of pollen in the samples. Sediments were transferred from one environment to another by vacuum filtration, collecting sample material onto a 10 µm stainless steel filter. Then, the proteolytic enzyme alcalase was used with the TRIS buffer (pH 8.2), while viscozyme and cellulase (cellulose blend) with acetate buffer (pH 4.8) were added. Before the Fenton reaction, samples were transferred into 200 ml of filtered Milli-Q water and cooled to 15-20 °C. During the reaction, the samples were kept within the temperature range 20-30 °C for at least 4 hours, then left to stand overnight at an ambient temperature. Next, the final density separation was performed, after which the samples were washed with 500 ml warm (+50°C) filtered Milli-Q water and 200 ml 50 % ethanol solution and then flushed from the filter into clean crimp bottles using 50 % ethanol solution. The samples were dried at 50 °C until the total sample evaporated and then the volume was adjusted to 5 mL volume.

Table S1. Found polymers in lakes Seksu, Pinku, Usmas. The microplastic concentration (particles·g^{-1}) throughout the core and corresponding age of the dated core layer. Abbreviations and explanations: PAN - polyacrylonitrile; Acrylates – combined group of derivative polymer of acrylic acid, SAN (styrene-acrylonitrile), ABS (acrylonitrile-butadiene-styrene), NBR (acrylonitrile butadiene rubber); Rubbers – combined group of BIIR (butyl rubber), BR (butadiene rubber), SR (synthetic rubber); PA - polyamide; PE - polyethylene; PES - polyester; PET – polyethylene terephthalate; BIO – combined group of polylactic acid (PLA) and polyhydroxybutyrate (PHB); POM - polyoxymethylen; PP - polypropylene; PS - polystyrene; PUR - polyurethane; PVA - polyvinyl acetate; PVC - polyvinyl chloride.

SEKSU			
**Polymer**	**Depth (cm)**	**Year**	**Concentration (particle·g^{-1})**
PAN	0-10	2019-2007	10.68
Acrylates			2.55
Rubbers			10.32
PA			6.92
PE			16.27
PES			1.70
PET			6.56
BIO			9.83
POM			0.61
PP			3.04
PS			12.26
PUR			10.08
PVA			7.41
PAN	10-15	2007-1997	10.68
Rubbers			1.58
PA			14.24
PE			8.70

PINKU			
**Polymer**	**Depth (cm)**	**Year**	**Concentration (particle·g^{-1})**
Rubbers	0 - 5	2019-2002	4.29
PE			1.68
PET			0.19
PUR			4.10
PVA			0.37
PA	5-10	2002-1953	0.05
PE			0.15
PES			0.15
PP			0.21
PUR			0.10
PVA			0.13
Rubbers	10-15	1953-1881	0.28
PA			0.84
PE			0.47
PES			1.45
BIO			1.17
PP			0.61

USMAS			
**Polymer**	**Depth (cm)**	**Year**	**Concentration (particle·g^{-1})**
Acrylates	0 - 5	2019-1997	0.08
Rubbers			1.90
PA			0.30
PE			0.38
PP			0.23
PS			0.15
PUR			0.38
PVA			0.46
PVC			0.23
PAN	10-15	1971-1949	0.06
PA			0.11
PES			0.22
PET			0.06
PP			0.17
PUR			1.06
PVA			0.39
PVC			0.17

PES			11.87
BIO			13.45
PP			8.70
PS			0.79
PUR			17.80
PVA			11.47
PVC			0.79
PAN	15 - 20	1997-1988	0.83
PA			1.00
PE			2.49
PES			0.33
PP			1.82
PS			0.33
PUR			4.64
PVA			0.83
PVC			0.17
PAN	20-25	1988-1979	0.52
Rubbers			5.35
PE			1.38
PES			0.86
PET			0.35
BIO			0.86
PS			3.45
PUR			4.49
PVA			6.21
PVC			0.35
PAN	25-30	1979-1969	0.15
PA			0.30

PUR			0.47
PVA			0.47
PVC			0.09
PA	15-20	1881-1813	1.36
PUR			0.47
PVA			0.63
PA	20-25	1813-1733	0.24
PE			0.18
PES			0.29
PET			0.03
BIO			0.05
PP			0.08

Rubbers			0.17
Rubbers	15-20	1949-1925	0.15
PA			0.15
PE			0.31
PES			0.08
PET			0.15
PP			0.08
PVA			0.15
Rubbers	20-25	1925-1900	0.15
PA			0.38
PE			0.61
PES			0.23
BIO			0.15
PP			0.31
PUR			0.46

PES			0.45
PET			0.30
BIO			0.15
PP			0.30
PUR			0.60
PA	30-35	1969-1959	1.29
PE			3.11
PES			0.78
PP			2.85
PS			0.52
PUR			1.04
PVA			4.40
PVC			0.26
PAN	35-40	1959-1949	0.26
Rubbers			0.52
PA			2.59
PE			3.63
PES			0.52
PS			2.07
PUR			0.26
PVA			5.96
PA	40-45	1949-1936	1.51
PAN			0.25
PE			1.76
PUR			0.25
PVA			1.26

Table S2. The total microplastic concentrations (particles·g^{-1}) in lakes Seksu, Pinku, Usmas. Concentrations throughout the core and average concentration, standard deviation (SD) are shown.

Lake	Depth (cm)	Concentration (particle g-1)	Average concentration (particle g-1)	SD
**SEKŠU**	0-10	98.22	33.99	40.76
	10-15	100.09		
	15 - 20	12.44		
	20-25	23.81		
	25-30	2.25		
	30-35	14.24		
	35-40	15.80		
	40-45	5.04		
**PINKU**	0 - 5	10.63	4.12	4.18
	5-10	0.80		
	10-15	5.84		
	15-20	2.46		
	20-25	0.86		
**USMAS**	0 - 5	4.11	2.47	1.25
	10-15	2.40		
	15-20	1.07		
	20-25	2.30		

Table S3. Output from principal component analysis (PCA): A - variable coordinates in nine principal components; B - quality of representation of the variables on factor map COS^2 (square *cosine*, *squared* coordinates); C - variable contribution in accounting for the variability in a given principal component (%). Abbreviations: MN – minor dimension, MJ – major dimension, PD – polymer density, DE – depth, DA – date, SH – shape, OM – organic matter, BD – dry bulk density, RBD – relative bulk density.

A. Variable coordinates

	PC1	PC2	PC3	PC4	PC5	PC6	PC7	PC8	PC9
MN	0.44895	0.73161	-0.48240	0.01853	-0.03553	0.02156	-0.00670	0.00667	-0.16832
MJ	-0.04847	0.75683	-0.05090	-0.02072	0.64055	0.01602	0.00966	-0.00187	0.10575
PD	-0.02124	0.16892	0.20776	-0.85480	-0.13455	0.42292	-0.01125	0.00742	0.00443
Depth	0.34337	-0.51940	-0.59538	-0.33720	0.28398	-0.18819	0.10833	0.12777	-0.00256
Date	-0.83503	0.19974	-0.06122	0.30450	-0.13769	0.34068	0.06282	0.16548	-0.00154
Shape	0.55885	0.28414	-0.56853	0.06435	-0.50910	0.01184	-0.00802	0.00928	0.14179
OM	-0.80586	-0.00085	-0.52890	-0.10649	-0.06075	-0.00795	0.19288	-0.13619	-0.00300
BD	0.77167	0.04656	0.51238	0.18476	-0.01373	0.19401	0.26039	-0.00772	-0.00071
RBD	0.30497	-0.45398	-0.43300	0.25647	0.25512	0.61135	-0.07386	-0.05770	-0.00239

B. Quality on the factor map COS2

	PC1	PC2	PC3	PC4	PC5	PC6	PC7	PC8	PC9
MN	0.202	0.535	0.233	<0.001	0.001	<0.001	<0.001	<0.001	0.028
MJ	0.002	0.573	0.003	<0.001	0.410	<0.001	<0.001	<0.001	0.011
PD	<0.001	0.029	0.043	0.731	0.018	0.179	<0.001	<0.001	<0.001
Depth	0.118	0.270	0.354	0.114	0.081	0.035	0.012	0.016	<0.001
Date	0.697	0.040	0.004	0.093	0.019	0.116	0.004	0.027	<0.001
Shape	0.312	0.081	0.323	0.004	0.259	<0.001	<0.001	<0.001	<0.001
OM	0.649	<0.001	0.280	0.011	0.004	<0.001	0.037	0.019	<0.001
BD	0.595	0.002	0.263	0.034	<0.001	0.038	0.068	<0.001	<0.001
RBD	0.093	0.206	0.187	0.066	0.065	0.374	0.005	0.003	<0.001

C. Variable contribution

	PC1	PC2	PC3	PC4	PC5	PC6	PC7	PC8	PC9
MN	7.550	30.846	13.772	0.033	0.147	0.063	0.035	0.068	47.487
MJ	0.088	33.009	0.153	0.041	47.853	0.035	0.074	0.005	18.742
PD	0.017	1.644	2.555	69.372	2.111	24.084	0.100	0.084	0.033
Depth	4.416	15.547	20.979	10.796	9.406	4.769	9.280	24.797	0.011
Date	26.118	2.299	0.222	8.803	2.211	15.628	3.120	41.594	0.004
Shape	11.698	4.653	19.130	0.393	30.228	0.019	0.051	0.131	33.698
OM	24.325	0.000	16.556	1.077	0.430	0.009	29.416	28.173	0.015
BD	22.305	0.125	15.537	3.241	0.022	5.068	53.611	0.091	0.001
RBD	3.484	11.877	11.096	6.245	7.591	50.326	4.314	5.058	0.010
